# Implementation mechanisms used in national efforts to improve community services to keep individuals with mental illness out of local jails

**DOI:** 10.21203/rs.3.rs-6007828/v1

**Published:** 2025-04-16

**Authors:** Niloofar Ramezani, Faye S. Taxman, Benjamin J. Mackey, Jill Viglione, Jennifer E. Johnson

**Affiliations:** Department of Biostatistics, Virginia Commonwealth University. 830 East Main Street, Box 980032, Richmond, VA , USA.; Schar School of Policy & Government, Center for Advancing Correctional Excellence, George Mason University, 4400 University Drive, Fairfax, VA, USA.; Center for Advancing Correctional Excellence, Schar School of Policy & Government, George Mason University, 4400 University Drive, 6D3, Fairfax, VA, USA.; Department of Criminal Justice, University of Central Florida, 12494 University Blvd., Orlando, FL, USA.; Charles Stewart Mott Department of Public Health, Michigan State University, 200 East 1^st^ Street, Flint, MI, USA.

**Keywords:** Implementation Mechanisms, Relationship-Building, Performance Monitoring, Interagency Coordination, Justice-Health Reforms

## Abstract

**Background:**

Little is known about effective implementation processes by which counties can improve community services to keep people with mental illness and substance use disorders out of the local jails. This study examines hypothesized implementation mechanisms (relationship building, performance monitoring, interagency coordination, capacity building, and infrastructure programming) as predictors of outcomes (improved community services) and as mediators of the effects of a national implementation intervention (Stepping Up [SU]), on community services.

**Methods:**

A survey was conducted of mental health, substance use, jail, and probation administrators in 519 U.S. counties, of which 328 counties participated in a national jail reform effort (SU). Survey data were combined with descriptive data from the U.S. Census Bureau. Predictors included hypothesized implementation mechanisms (performance monitoring, interagency coordination teams, creating integrated systems of care, capacity building, relationship building, and quality programming). Covariates included county sociodemographic characteristics (e.g., size of county, size of jail, etc.) and general county service characteristics (e.g., primary care physicians per capita, Medicaid expansion). Implementation outcomes included number of evidence-based practices (EBPs) and evidence-based mental health treatments (MH-EBTs) for individuals with mental illness involved with justice systems. Multilevel regression analyses examined cross-sectional: (1) effects of Stepping Up on outcomes; (2) effects of implementation mechanisms on implementation outcomes; and (3) implementation mechanisms as mediators of the effects of Stepping up on implementation outcomes.

**Findings:**

SU was found to significantly predict the number of EBPs and MH-EBTs controlling for various demographic characteristics of the counties. When implementation mechanisms were added to these models, SU is no longer statistically significant. Instead, two implementation mechanisms (performance monitoring and interagency coordination) and Medicaid funding significantly predicted the availability of both EBP and/or MH-EBT. Other factors that predicted MH-EBTs include relationship building size of the county, rate of primary care physicians, rate of MH providers in the county, and jail population size. Mediation models found that SU significantly predicted these evidence-based outcomes through implementation mechanisms except interagency coordination.

**Conclusions:**

Little is known about the implementation mechanisms to decarcerate and build programming for MH services in a county. SU is an important attribute to facilitate reform both directly and indirectly through implementation mechanisms. Counties can benefit from use of relationship building activities to advance policy and service reform efforts, identifying performance metrics of their system, and having infrastructure available to improve the availability of EBPs. Overall, policy changes are possible, but an emphasis should be on strategies that increase the availability of EBPs and MH-EBTs.

## INTRODUCTION

With more than 10 million admissions to and releases from local jails in the U.S. each year, jails confine people that are typically unsentenced or serving short sentences, they are run by counties and cities, and with a median length of stay of 72 hours (Torrey, et al., 2010). Local jails in the U.S. often house people with mental health (MH) disorders due to the dearth of available MH treatment services and facilities in the community (Torrey et al., 2010;xx, et al., 2021). People with MH conditions experience jail incarceration more often, spend more time in jail, and are more likely to return to jail than individuals without mental illness (Wrenn et al., 2018). Reducing the use of jail for people with mental health problems is a complex policy issue, often requiring interagency efforts that impact jail intake procedures, resources needed for programs and services in the jail and community, support from agencies ranging from law enforcement to judicial actors to community groups, and coordination between in-jail and community-based treatment services. Each area is a challenge in itself, but collectively decarcation policies and practices require a commitment to identify the individuals with MH issues and divert them from jail using community-based services. It is important to have sufficient community-based treatment services for decarceration efforts to be successful.

xx and colleagues (2023) documented 18 recommended evidence-based practices and 42 MH treatments to divert people with mental illness from jail and/or provide treatment to improve the individuals’ functioning. Each is offered in only 22–43% of U. S. counties. The low uptake of these practices and treatments attests to the difficulties in improving service delivery as a tool to decarcerate local jails.

Implementation science is increasingly prioritizing the study of implementation mechanisms to better understand efforts to improve services work. Relatively little is known in implementation science generally, and even less is known about implementation mechanisms at the intersection of community MH services and local jails and probation systems. This manuscript reports on a planned analysis of implementation mechanisms in the Implementation Mechanisms (I.M.) of Behavioral Health (xx, et al., 2021) study. These include relationship building among agencies on a policy team, performance monitoring (i.e., deciding on key agency metrics and tracking them over time to gauge service improvement efforts), interagency coordination (such as regular meetings of county mental health, substance use, jail, and probation leaders, sharing resources, etc.). Other related mechanisms are capacity building by hiring staff to augment planning, programs, and analyses, and infrastructure programming to build quality services.

This paper examines (1) the effects of Stepping Up participation on outcomes; (2) the effects of implementation mechanisms on outcomes; and (3) implementation mechanisms as mediators of the effects of Stepping up on outcomes. Stepping Up is a national implementation approach adopted by over 582 counties in the U.S. to alter how they use their local jails for people with mental illness (Haneberg et al., 2017). The goal is to reduce the size of local jail populations by building the capacity of county service systems to provide evidence-based mental health and substance use care. Stepping Up provides a series of recommended steps for counties to follow that are typical strategies used in criminal legal system reforms. To assist counties, Stepping Up organizations provide online technical assistance including webinars, resource materials, toolkits, mentoring sites, and specialized assistance for performance metric development. The on-line technical assistance focuses primarily on interagency teams, performance monitoring, and MH treatment services. Stepping Up is open to any county that desires to join the initiative.

## METHODS

This study investigates the participation in Stepping Up, county characteristics, implementation mechanisms, and organizational factors that might explain the availability of EBPs and MH-EBTs in a jurisdiction. The list of EBPs and MH-EBTs is derived from a consensus-building exercise conducted as part of this study (see xx et al., 2023).

### Data Source

The main data for this research came from a survey of 519 U.S. counties conducted between 2020 and 2022. This survey was the first wave of a 5-year study aimed at comparing implementation mechanisms and outcomes between counties that joined Stepping Up and matched pair counties that did not join this initiative (xx et al., 2021; xxezani et al., 2020). 950 counties were contacted (475 Stepping Up counties and 475 control counties), from which a total of 519 counties completed the baseline survey questionnaire, which is a 55% response rate at the county level. This is a relatively high response rate for organizational survey-related research (Fincham, 2008; Story & Tait, 2019). The response rate for Stepping Up counties was 69%, while 40% of the control counties responded to this survey.

Predictors used in this study are from a dataset previously compiled by this research team (see xxezani et al., 2022 and xx, et al., 2021 for a description of the survey and extant data sets). R (R Core Team, 2021) was used to perform the predictive models. A significance level of 0.05 was used for the analysis.

### Assessments

#### Hypothesized implementation mechanisms.

The implementation mechanisms examined in our study, shown in Table 1, consisted of five scales developed using factor analysis and each had a Cronbach alpha greater than 0.7 (xxezani et al., Paper in progress) including (1) relationship-building among agencies to coordinate policies and programs; (2) performance metrics monitoring to assess efforts to improve service delivery and decarceration; (3) interagency coordination of efforts to provide services and decarcerate; (4) capacity building by hiring staff to augment planning, programs, and analyses; and (5) infrastructure programming to build quality services through specialized units, decision matrices for program placement, programs for higher risk individuals, and targeting specific needs. Although the original design was to include four mechanisms, we are using the five that were derived from factor analyses of the original survey questions (see xx, et al., 2021). That is, factor analyses revealed another a cohesive set of mechanisms that counties used to advance their reforms. Table 1 provides a definition for each strategy and descriptive statistics.

#### Covariates.

County-level socio-demographic and general service variables were used as covariates in all analyses. They were selected based on the literature and previous empirical studies of their relationship with the evidence-based programs and treatment-related outcome variables (see xxezani, et al., 2023). Backward variable selection method was used to narrow down the variables used in the final models. We checked for multicollinearity among variables using a correlation matrix and the variance inflation factor index. We excluded variables that were highly correlated with the rest of the variables (Montgomery & Friedman, 1993) after ensuring that their variance is explained through other variables included in the models.

Table 2 summarizes the county characteristics used as covariates in our models. Ten county sociodemographic characteristics were used, including a categorical variable for county size (< 250,000, between 250,000 and 750,000, and over 750,000 county population, indicating county sizes small, medium, and large, respectively), whether the county was located in a rural geographic region, percentage of Hispanic and Black population in the county, per capita county jail population size, MH provider rate (psychiatrists, psychologists, counselors, and social workers), primary care physician rate, whether agencies in the county received Medicaid funding, and whether the county was a designated medically underserved county.

#### Outcomes.

Implementation outcomes in this study included: (1) number of evidence-based practices (EBP) and (2) number of evidence-based treatments (MH-EBT) for individuals with mental illness involved with justice systems. Definitions are provided in Table 1 below with descriptive statistics.

#### Stepping Up status.

Stepping Up status was derived from the Stepping Up website listing all counties that have signed a resolution to be part of the initiative at the time of data collection.

### Statistical Analysis

Statistical analyses were conducted in R (R Core Team, 2021). Multilevel regression (i.e. hierarchical linear model [HLM]) and mediation analyses were employed to examine: (1) the effects of Stepping Up on various outcomes; (2) effects of implementation mechanisms on each outcome; and, (3) (3) implementation mechanisms as mediators of the effects of Stepping up on implementation outcomes. As recommended by Raudenbush and Bryk (2002), we calculated the intraclass correlation coefficient (ICC), reflecting counties nested within states, as an initial step to determine whether performing multilevel models was necessary. ICC values ranged around .1 and, as recommended in the work by Lee (2000) for an interclass correlation of .1 and above, we used HLM nesting counties within states. Analyses employed a two-level HLM to account for the clustering of counties within states, addressing potential correlations among responses within states, and the hierarchical nature of our data. Because EBPs and MH-EBPs consisted of count measures, which followed a Poisson distribution, generalized hierarchical linear models (GHLM) using the Poisson family and a logarithmic link function, adding the state clustering effect, were used (Gelman & Hill, 2006). To investigate the potential mediation effects, mediation analyses were employed using a Sobel test (Sobel, 1982).

## RESULTS

The average number of EBPs was 13.0 out of 18 and the average number of MH EBPs was 29.6 of 42 (see Table 1). The implementation strategies are also presented in Table 1. Nearly 70 percent of counties reported the use of *relationship building* mechanisms with other relevant agencies; this mechanism had a comparatively high mean response compared to other implementation mechanisms. Around 38 percent of the counties reported using *performance monitoring* mechanisms, 38 percent reported the use of *interagency coordination across agencies,* 43.5 percent reported using *capacity-building efforts,* and 51.4 percent reported the use of *infrastructure* programming mechanisms to provide quality services.

### Impact of Stepping Up on EBP and MH-EBTs

Stepping Up status significantly predicted both more EBPs and more MH EBPs after controlling for rurality, county size, medically underserved area, percent of Hispanic and Black population, primary care physicians’ rate, jail population per capita, MH provider rate, and Medicaid funding for services in the counties (Table 3). Table 3 shows that being a Stepping Up county is positively associated with EBPs and MH EBPs (p < 0.001 and 0.018502, respectively). Detailed models with all the coefficients can be found in Supplemental Table S2.

### Impact of Implementation Mechanisms on EBP and MH-EBTs

Table 4 shows how implementation mechanisms predict implementation outcomes (EBPs and MH EBPs) after controlling for rurality, county size, medically underserved area, percent of Hispanic and Black population, primary care physicians’ rate, jail population per capita, MH provider rate, and Medicaid funding for services in the counties. Performance monitoring (p < 0.001) and interagency coordination (p< 0.001) both predicted more EBPs and MH-EBTs. Relationship building only predicted more MH EBPs (p < .001). Neither infrastructure programming nor capacity building predicted number of EBPs or MH-EBTs in these covariate-adjusted models. Detailed models with all the coefficients can be found in Supplemental Table S1.

### Mediation Analysis

#### Step 1: Exploratory analyses.

When including both Stepping Up status and implementation mechanisms in the covariate-adjusted models, the direct effect of Stepping Up status on EBPs and MH-EBTs is no longer significant (see Table 5). Relationships building implementation mechanisms and EPBs/MH-EBTs were the same as when Stepping Up status was not included as a predictor in the models. Specifically, in predicting EBPs, performance monitoring (p = 0.001) and interagency coordination (p < 0.00) are both associated with having more EBPs, while Stepping Up status is no longer significant (p = 0.73). In predicting MH-EBTs, relationship building strategies (p < 0.001), performance monitoring (p < 0.001) and interagency coordination (p < 0.001) are all positively associated with MH-EBPs while Stepping Up status is no longer significant (p = 0.13). Infrastructure programming and capacity building still did not predict number of EBPs or MH-EBTs. Detailed models with all the coefficients can be found in Supplemental Table S3.

These results suggest that Stepping Up status may have an indirect on the EBPs and MH-EBPs through some implementation mechanisms. Therefore, mediation analyses were performed to test this indirect effect.

#### Step 2.

**Figure 1:**
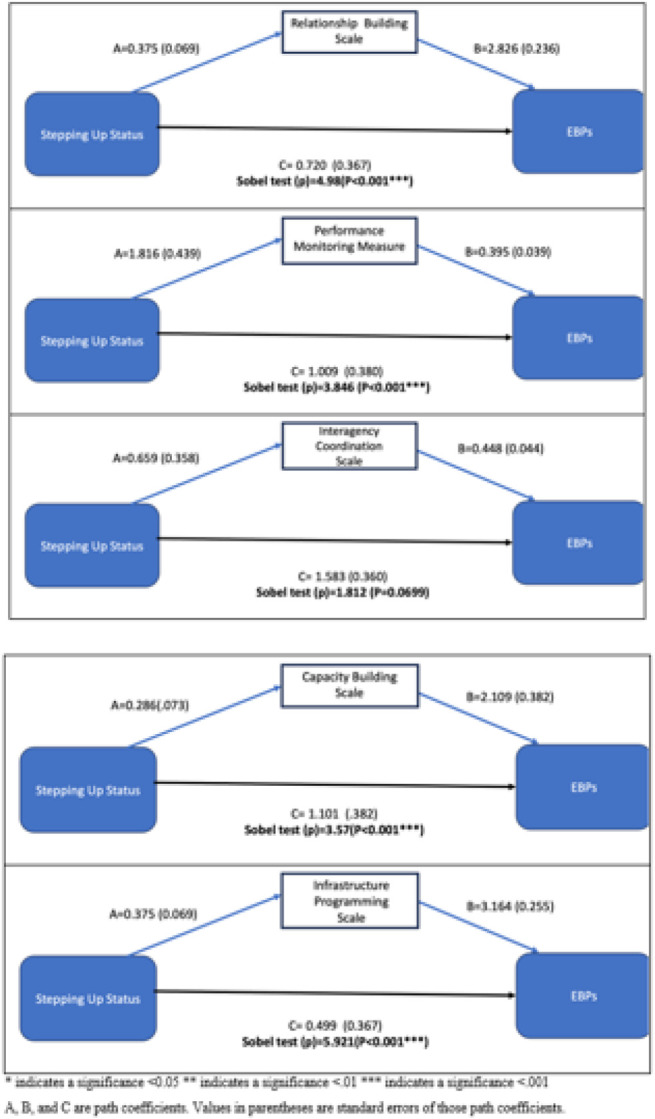
Mediation models when predicting number of EBPs

When predicting the EBPs via each implementation mechanism (see Fig. 1 for relationship building, performance monitoring, interagency coordination, capacity building, and infrastructure programming mechanisms), Stepping Up status had a significant indirect/mediated effect on the legal EBPs via four scales. The indirect effect between Stepping Up status and EBPs was significant via the relationship building scale (p < 0.001), performance monitoring scale (p < 0.001), infrastructure programming scale (p < 0.001), and capacity building scale (p < 0.001). This indirect effect was approaching statistical significance via the interagency coordination scale (p-value = 0.07).

**Figure 2:**
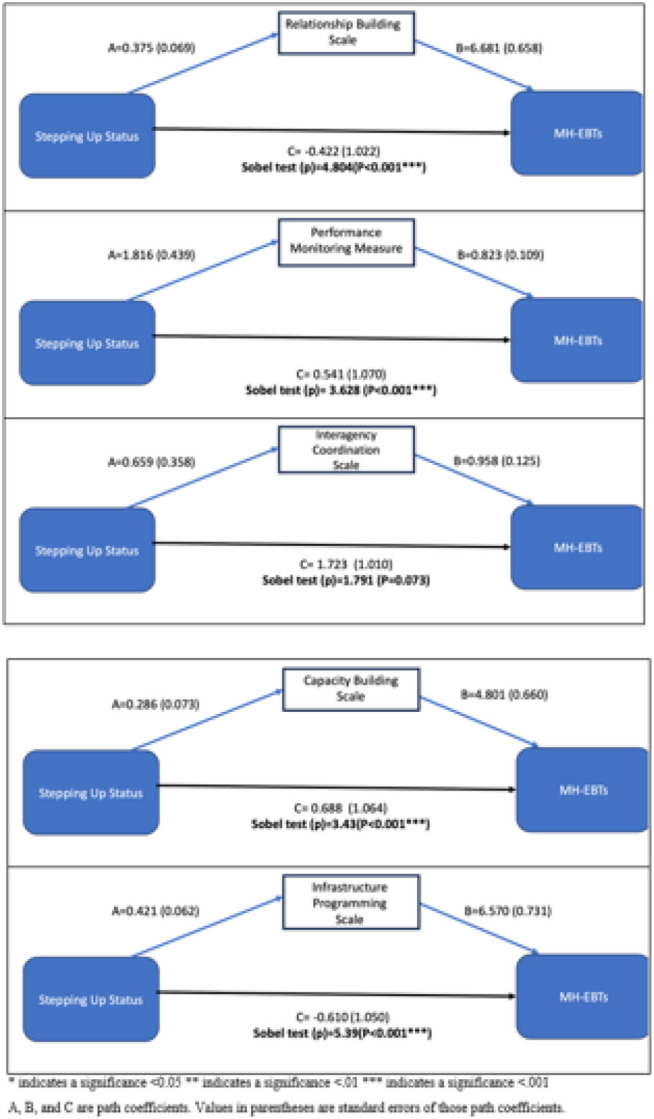
Mediation models when predicting number of MH-EBTs

When predicting the MH EBPs via implementation mechanism (see Fig. 2), Stepping Up status had a significant indirect/mediated effect on the MH-EBTs like EBPs. The indirect effect between Stepping Up status and MH-EBPs was significantly mediated via the relationship building scale, performance monitoring measure (p < 0.001), capacity building scale (p-value < 0.001), and infrastructure programming implementation mechanisms (p-value < 0.001). This indirect effect was not statistically significant through the interagency coordination scale (p-value = 0.07).

### Demographic Variables Associated with Implementation Outcomes

The results of two HLM regression models, which show results for covariates in models predicting the number of EBPs and MH-EBTs, are presented in Supplementary Table S3. Medicaid funding for services (z-score = 2.79, p < 0.01) was the only variable positively associated number of EBPs. Significant predictors of the number of MH-EBTs were: county size (medium vs. large; z = 2.54, p = 0.01), primary care physicians’ rate (z-score = 3.93, p < 0.001), jail population per capita (z-score = 1.9814, p< 0.05), MH provider rate (z-score = −2.67, p < 0.01), and Medicaid funding for services (z-score = 6.0005, p < 0.001).

## DISCUSSION

Over 582 counties in 40 states have joined the Stepping Up Initiative, one of the largest reform efforts devoted to decarceration of individuals with MH disorders from jail and increasing the capacity in the community for providing MH services. This analysis demonstrated that, controlling for covariates, use of performance monitoring, interagency coordination and relationship building implementation mechanisms predicted having more EBPs and/or mental health EBTs in counties. Furthermore, controlling for covariates, being in Stepping Up was associated with having more EBPs and mental health EBTs in a county. Finally, the effects of Stepping Up on the number of EBPs and mental health EBTs was mediated by all mechanisms except for interagency coordination. SU is a robust predictor of both outcomes; more importantly the implementation mechanisms are more vibrant and predictive in the presence of SU counties.

Intriguingly, relationship-building was not found to be a significant implementation mechanism for EBPs, contradicting some prior evidence (xx et al., 2024a). This may be due to the inclusion of the interagency coordination and performance monitoring scales in the models, both which have larger z scores than the other implementation mechanisms Essentially, both performance monitoring and interagency coordination are the ideal product of relationship building, as they reflect the achievement of functional policy teams. Thus, relationship-building may have served as a proxy for actual relationships in prior analyses (xx et al., 2024a). From an implementation perspective, relationship building through interagency policy teams are critical to make a commitment to reform, and the way the teams operate are important to making strides in difficult policy arenas (xx et al., 2024a; 2024b)—particularly to address the system issues that affect screening, identification, and placement of individuals with need in appropriate services. Policy teams do not need to have consensus on goals, but they do need to have sufficient infrastructure to support their initiatives (xx et al., 2024b).

It is apparent that the way in which counties pursue a change in policy and practice identifies key mechanisms used to change the systems, as well as largely defines the difference between Stepping Up and non-Stepping Up counties. The counties that join Stepping Up appear to be different than other counties since Stepping Up has a direct effect on EBPs and MH-EBTs after controlling for county characteristics. However, when the implementation mechanisms are added to the models, Stepping Up is no longer statistically significant. But mediation analyses found that Stepping Up counties for the most part contribute to a stronger working relationship among team members, having performance metrics to gauge progress, more attention to infrastructure, and having staff resources devoted to building capacity. This is true for both outcomes: EBPs and MH-EBTs. Interesting, interagency coordination of services does not appear to be important in advancing the use of EBPs or MH-EBTs. The mediation effect illustrates the importance of the Stepping Up reform framework in impacting policies and practices; it means that counties that pursue these implementation strategies may be more likely to expand their use of EBPs and MH EBTs.

Few county-level socio-demographic characteristics were statistically significant predictors of the outcomes related to the EBPs and MH-EBTs. Counties that received Medicaid funding were more likely to have a higher number of EBPs and MH-EBTs. Medium-sized counties had a higher number of MH-EBTs compared to large counties. Smaller counties had fewer MH-EBTs compared to large counties even though the difference was not statistically significant. No other socio-demographic variables predicted the differences among counties in terms of the availability of EBPs or MH-EBTs. Overall, these findings about county characteristics are surprising because we would have expected county size, being part of the medically underserved counties, and some population characteristics to predict various outcomes.

Study strengths include attention to the reform mechanisms that can impact EBPs and MH-EBTs outcomes which helps to identify forward processes that can affect building services in the community. Limitations of the study include its cross-sectional design, which precludes causal conclusions, and the timeframe of data collection, which occurred during COVID-19 when policy teamwork was more difficult due to lack of in-person events. It is possible that some counties were unable to work on decarceration efforts during this time. The survey was completed by a representative of the county, and it is possible that person did not know about the efforts other agencies were engaged with. This is also cross-sectional data and thus inferences regarding causal direction (i.e., whether increased use of implementation strategies leads to more EBPs/MH-EBTs or vice-versa) cannot be drawn.

## CONCLUSION

Effective implementation mechanisms can impact the number of EBPs and MH-EBTs available, particularly for counties that partake in Stepping Up. This study illustrates that enhanced treatment and practice reforms are feasible, but the internal mechanics of how implementation is pursued affects progress. And, counties that join SU are more likely to be effective in the implementation mechanisms to affect change. That socio-demographic characteristics of the county were less important than *how* counties pursue reforms. Instead, counties that are part of SU appear to implicitly have the support of leadership to engage in change efforts to effectuate decarceration. This suggests technical assistance to facilitate better reform strategies is a wise strategy, particularly when leadership supports reform efforts such as joining SU. Focusing on implementation strategies can help counties overcome any limitations due to the nature and size of their county population, availability of funding, and availability of sufficient services and staffing. In particular, performance metrics and interagency coordination are key implementation mechanisms that affect the number of EBPs and MH-EBTs since they are efforts to work across agencies to enhance efforts to reduce the use of jail for people with mental health issues. Efforts like Stepping Up strengthen reform efforts by providing a strategic path forward, even in counties that need to build relationships, infrastructure, and make a commitment to quality services.

## Figures and Tables

**Table 1.** Descriptive Statistics of Response and Implementation Mechanisms

Dependent Variables: Continuous and Count Response Variables							
		Total		SU		Non- SU	
		Mean	S. D	Mean	S. D	Mean	S.D
Number of EBPs (of 18)	Number of criminal justice evidence-based programs/policies available in a county	12.97	4.41	13.66	4.050	11.77	4.75
Number of MH-EBTs (of 42)	Number of evidence-based MH treatments available	29.54	11.69	30.34	11.16	28.15	12.49
Predictor Variables		Total		SU		Control	
Implementation Strategy Scales		Mean	SD	Mean	SD	Mean	SD
Relationship Building	*Policy/Team Relationship Building Scale:* Agencies build relationships through sharing information, collaborative problem-solving, using or providing technical assistance to improve cross-agency assessment and screening, staff training, etc.	2.54	0.75	2.67	0.71	2.30	0.76
Performance Monitoring Measure	Respondent indicates that their agency tracks measures for populations with mental illness: (1) number of people with mental illnesses who are booked into jail, (2) average length of stay in jail, (3) percent linked to community-based treatment after release from jail; and (4) return to jail rate (i.e., recidivism). and (5) length of stay post jail treatment	4.46	4.67	5.10	4.86	3.28	4.04
Interagency Coordination	*Interagency Coordination Scale:* Level of collaboration including sharing information, developing eligibility criteria, pooling funding or staff, sharing oversight, implementing cross-training, and developing protocols.	10.88	3.91	11.12	3.81	10.46	4.04
Capacity Building	*Capacity Building Scale:* Improve service capacity of different agencies within counties by hiring experts, therapists, psychiatrists, peer support specialists, boundary spanner working in more than one agency, and/or county coordinator, and obtaining behavioral health licensing.	2.06	0.78	2.16	0.77	1.88	0.75
Infrastructure Programming (Quality)	*Infrastructure Programming Scale:* Agencies used strategies to expand services for justice-involved individuals with mental illness and improve mental health or substance use services for justice-involved individuals.	2.16	0.63	2.34	0.66	1.90	0.67

**Table 2.** Characteristics of the Counties

	Total		SU		Control	
Continuous predictors	Mean	SD	Mean	SD	Mean	SD
Jail Pop Per Capita	0.003	0.002	0.003	0.002	0.003	0.002
%Hispanic & Black	19.42	18.23	21.19	19.19	16.38	16.04
Primary Care Physicians Rate	68.23	30.20	70.87	30.61	63.61	28.99
MH Providers Rate	186.35	135.94	202.71	148.03	157.99	106.50
Ratio of Agencies in the County Receiving Medicaid Funding	0.43	0.45	.42	0.44	0.45	0.46
Total Number of Staff	142.81	369.34	159.22	404.96	113.60	294.47
Categorical predictors	Frequency	Proportion	Frequency	Proportion	Frequency	Proportion
Rural County	Rural: 88	0.170	40	0.122	48	0.251
	Urban: 431	0.830	288	0.878	143	0.749
Large County	48	0.092	39	0.119	9	0.047
Medium County	103	0.198	75	0.229	28	0.147
Small County	368	0.709	214	0.652	154	0.806
Medically Underserved (MU)	MU: 103	0.206	53	0.165	50	0.279
	Not MU: 398	0.794	269	0.835	129	0.721
Stepping Up (SU)	SU: 328	0.632	-	-	-	-
	Not SU: 191	0.368				

**Table 3.** HLM models to Predict EBPs and MH-EBTs from SU Status

	Model 1: EBPs (18 programs/practice) 435 counties in 40 states				Model 2: MH-EBTs (42 MH treatments) 435 counties in 40 states			
	Estimate	SE	Z	P	Estimate	SE	Z	P
SU Status	0.0904	0.0293	3.0888	0.001**	0.046	0.0195	2.3564	0.02*

These models controlled for Rurality, county Size, medically underserved area, Percent of Hispanic and Black population, primary care physicians’ rate, jail population per capita, MH provider rate, and Medicaid funding for services

* indicates a significance <0.05

** indicates a significance <.01

*** indicates a significance <.001

Note: Detailed models with all the coefficients can be found in Supplemental Table S2

**Table 4.** HLM models to Predict EBPs and MH-EBTs from Implementation Mechanisms

	Model 1: EBPs (18 programs/practice) 435 counties in 40 states				Model 2: MH-EBTs (42 MH treatments) 435 counties in 40 states			
	Estimate	SE	Z	P	Estimate	SE	Z	P
Relationship building	0.045	0.032	1.41	0.16	0.080	0.022	3.6756	<0.001***
Performance monitoring	0.011	0.003	3.26	0.001**	0.011	0.002	4.8601	<0.001***
Interagency coordination	0.018	0.004	4.14	<0.001***	0.016	0.003	5.1909	<0.001***
Infrastructure programming	0.052	0.036	1.47	0.15	0.022	0.024	0.9124	0.36
Capacity building	0.028	0.023	1.19	0.23	0.027	0.016	1.726	0.08

These models controlled for rurality, county Size, medically underserved area, Percent of Hispanic and Black population, primary care physicians’ rate, jail population per capita, MH provider rate, and Medicaid funding for services

* indicates a significance <0.05

** indicates a significance <.01

*** indicates a significance <.001

Note: Detailed models with all the coefficients can be found in Supplemental Table S1

**Table 5.** Full HLM models to Predict EBPs and MH-EBTs from SU Status and Implementation Mechanisms

	Model 1: EBPs (18 programs/practice) 435 counties in 40 states				Model 2: MH-EBTs (42 MH treatments) 435 counties in 40 states			
	Estimate	SE	Z	P	Estimate	SE	Z	P
SU Status	0.011	0.032	0.340	0.73	−0.033	0.022	−1.532	0.12
Relationship building	0.045	0.032	1.385	0.17	0.082	0.022	3.763	<0.001***
Performance monitoring measure	0.011	0.003	3.228	0.001***	0.011	0.002	4.959	<0.001***
Interagency coordination	0.018	0.004	4.150	<0.001***	0.015	0.003	5.024	<0.001***
Infrastructure programming	0.051	0.036	1.405	0.16	0.027	0.024	1.103	0.27
Capacity building	0.027	0.023	1.188	0.23	0.028	0.016	1.747	0.08

These models controlled for Rurality, county Size, medically underserved area, Percent of Hispanic and Black population, primary care physicians’ rate, jail population per capita, MH provider rate, and Medicaid funding for services

* indicates a significance <0.05

** indicates a significance <.01

*** indicates a significance <.001

Note: Detailed models with all the coefficients can be found in Supplemental Table S2

## Abbreviations

EBP: Evidence-Based Policy and Practices
MH-EBT: Mental Health Evidence-based Treatments
SU: Stepping Up

## References

[1] Cuellar A. E., xxezani N., xx J. E., Breno A., & xx F. S. (2021). Drivers of County Engagement in Criminal Justice-Behavioral Health Initiatives, Psychiatric Services. Published Online: 13 Oct 2021 https://doi-org.mutex.gmu.edu/10.1176/appi.ps.202100485 10.1176/appi.ps.202100485 PMC9005561 34644126

[2] Cheng C., Spiegelman D. & Li F. Estimating the natural indirect effect and the mediation proportion via the product method. BMC Med Res Methodol 21, 253 (2021). 10.1186/s12874-021-01425-4 PMC8606099 34800985

[3] Fincham J. E. (2008). Response Rates and Responsiveness for Surveys, Standards, and the Journal. American Journal of Pharmaceutical Education, 72(2), 43. 10.5688/aj72024 18483608 PMC2384218

[4] Gelman A., Hill J. (2006). Data analysis using regression and multilevel/hierarchical models. Cambridge University Press.

[5] Haneberg R., Fabelo T., Osher F., & Thompson M. (2017). Reducing the number of people with mental illnesses in jail: Six questions county leaders need to ask. The Stepping Up Initiative. https://csgjusticecenter.org/wp-content/uploads/2020/02/Reducing-the-Number-of-People-with-Mental-Illnesses-in-Jail_Six-Questions.pdf

[6] xxJ. E., xxezaniN., ViglioneJ., HailemariamM., xxF. S. (2023). Recommended MH practices for individuals interacting with police, court, jail, probation, and parole in the U.S. Psychiatric Services. https://doi-org.mutex.gmu.edu/10.1176/appi.ps.2023002910.1176/appi.ps.2023002937933131

[7] xx J. E., Viglione J., xxezani N. Protocol for a quasi-experimental, 950 county study examining implementation outcomes and mechanisms of Stepping Up, a national policy effort to improve MH and substance use services for justice-involved individuals. Implementation Sci 16, 31 (2021). 10.1186/s13012-021-01095-2 PMC8006626 33781294

[8] Lee V. E. (2000). Using hierarchical linear modeling to study social contexts: The case of school effects. Educational Psychologist, 35(2), 125–141.

[9] xx B. J.,& xx F. S. (2023). Implementing jail reform: The approaches counties use to alter local jails. In Rudes D. S., Kras K., Carter T. J., & Armstrong G. (Eds.), Handbook on prisons and jails (pp. 345–363). Routledge. 10.4324/9781003374893-28

[10] xx B. J., xxezani N., Viglione J., Thurman T., xx J. E., & xx F. S. (2024a). Implementing reform: Approaches to alter the use of local jail for people with behavioral health conditions. International Journal of Offender Therapy and Comparative Criminology. Advance online publication. 10.1177/0306624X241294136 PMC12081762 39548799

[11] xx B. J., xx J. E., xxezani N., Hailemariam M., Rosen R. K., Thurman T., Viglione J., & xx F. S. (2024b). The who, what, and how of interagency criminal justice-behavioral health teams: Developing and sustaining collaborations. Criminal Justice & Behavior, 52(1), 61–78. 10.1177/0093854824128039. 39822735 PMC11737727

[12] Montgomery D. C., & Friedman D. J. (1993). Prediction using regression models with multicollinear predictor variables. IIE transactions, 25(3), 73–85.

[13] Powell B. J., Waltz T. J., Chinman M. J. A refined compilation of implementation strategies: results from the Expert Recommendations for Implementing Change (ERIC) project. Implementation Sci 10, 21 (2015). 10.1186/s13012-015-0209-1 PMC4328074 25889199

[14] xxezani N., Breno A.J., xx B.J. (2022). The relationship between community public health, behavioral health service accessibility, and mass incarceration. BMC Health Serv Res 22, 966 (2022). 10.1186/s12913-022-08306-6 35906627 PMC9336014

[15] xxezani N., Breno A., Viglione J., xx B., Cuellar A. E., Chase A., … & xx F. S. (2020, August). Multilevel Matching in Natural Experimental Studies: Application to Stepping up Counties. In Proceedings. American Statistical Association. Annual Meeting (Vol. 2020, p. 2408). NIH Public Access. PMC8035050 33841051

[16] xxezani N., Clark K., xx J. E., xx F. S. (2025). Measuring Implementation Strategies for Justice-Health Initiatives. To be submitted to Implementation Research and Practice.

[17] xxezani N., Hailemariam M., Breno A.J. xx B. J., Cuellar A. E., xx J. E., & xx F. S. (2023). Impact of County-level health infrastructure on participation in a reform effort to reduce the use of jail for individuals with mental health disorders. Health Justice 11, 27 10.1186/s40352-023-00226-9 37401987 PMC10318809

[18] Raudenbush S. W., Bryk A. S. (2002). Hierarchical linear models: Applications and data analysis methods (Vol. 1). Sage.

[19] R Core Team. (2017). R: A language and environment for statistical computing. R Foundation for Statistical Computing. http://www.R-project.org/

[20] Sobel M.E. (1982) Asymptotic Confidence Intervals for Indirect Effects in Structural Equation Models. Sociological Methodology, 13, 290–321. 10.2307/270723

[21] Story D.A. & Tait A.R. (2019). Survey Research. Anesthesiology. Feb;130(2):192–202. doi:10.1097/ALN.0000000000002436. 30688782

[22] xx F. S. (2024). Health Criminology: Addressing The Culture of Control. In Link N.W., Novisky M.A., & Fahmy C. Handbook on Contemporary Issues in Health, Crime and Punishment. Routledge.

[23] xx F. S., Shukla N., xxenzani N., Viglione J., & Johnston J. E. (2025). Exploring county-level resources influencing evidence-based practices and treatment uptake in behavioral health settings. Corrections, 1–16. 10.1080/23774657.2024.2447693 PMC12355971 40857444

[24] Torrey E. F., Kennard A. D., Eslinger D., Lamb R., & Pavle J. (2010). More Mentally Ill Persons Are in Jails and Prisons Than Hospitals: A Survey of the States. U.S. Department of Justice, Office of Justice Programs. https://www.ojp.gov/ncjrs/virtual-library/abstracts/more-mentally-ill-persons-are-jails-and-prisons-hospitals-survey

[25] Wrenn G, McGregor B, Munetz M. The Fierce Urgency of Now: Improving Outcomes for Justice-Involved People With Serious Mental Illness and Substance Misuse. Psychiatr Serv. 2018 Jul 1;69(7):829–831. doi: 10.1176/appi.ps.201700420. 29656711

